# *ZNF280BY *and *ZNF280AY*: autosome derived Y-chromosome gene families in *Bovidae*

**DOI:** 10.1186/1471-2164-12-13

**Published:** 2011-01-07

**Authors:** Yang Yang, Ti-Cheng Chang, Hiroshi Yasue, Arvind K Bharti, Ernest F Retzel, Wan-Sheng Liu

**Affiliations:** 1 Department of Dairy and Animal Science, The Center for Reproductive Biology and Health (CRBH), College of Agricultural Sciences, The Pennsylvania State University, University Park, PA 16802, USA; 2 The Integrative Biosciences Program, Bioinformatics and Genomics Option, The Huck Institute of Life Sciences, The Pennsylvania State University, University Park, PA 16802, USA; 3 National Institute of Agrobiological Sciences, Tsukuba, Ibaraki, 305-0901, Japan; 4 National Center for Genome Resources, Santa Fe, NM 87505, USA; 5 Current address: Department of Pathology and Laboratory Medicine, School of Medicine, University of North Carolina-Chapel Hill, Chapel Hill, NC 27599, USA

## Abstract

**Background:**

Recent progress in exploring the Y-chromosome gene content in humans, mice and cats have suggested that "autosome-to-Y" transposition of the male fertility genes is a recurrent theme during the mammalian Y-chromosome evolution. These transpositions are lineage-dependent. The purpose of this study is to investigate the lineage-specific Y-chromosome genes in bovid.

**Results:**

We took a direct testis cDNA selection strategy and discovered two novel gene families, *ZNF280BY *and *ZNF280AY*, on the bovine (*Bos taurus*) Y-chromosome (BTAY), which originated from the transposition of a gene block on the bovine chromosome 17 (BTA17) and subsequently amplified. Approximately 130 active *ZNF280BY *loci (and ~240 pseudogenes) and ~130 pseudogenized *ZNF280AY *copies are present over the majority of the male-specific region (MSY). Phylogenetic analysis indicated that both gene families fit with the "birth-and-death" model of evolution. The active *ZNF280BY *loci share high sequence similarity and comprise three major genomic structures, resulted from insertions/deletions (indels). Assembly of a 1.2 Mb BTAY sequence in the MSY ampliconic region demonstrated that *ZNF280BY *and *ZNF280AY*, together with *HSFY *and *TSPY *families, constitute the major elements within the repeat units. The *ZNF280BY *gene family was found to express in different developmental stages of testis with sense RNA detected in all cell types of the seminiferous tubules while the antisense RNA detected only in the spermatids. Deep sequencing of the selected cDNAs revealed that different loci of *ZNF280BY *were differentially expressed up to 60-fold. Interestingly, different copies of the *ZNF280AY *pseudogenes were also found to differentially express up to 10-fold. However, expression level of the *ZNF280AY *pseudogenes was almost 6-fold lower than that of the *ZNF280BY *genes. *ZNF280BY *and *ZNF280AY *gene families are present in bovid, but absent in other mammalian lineages.

**Conclusions:**

*ZNF280BY *and *ZNF280AY *are lineage-specific, multi-copy Y-gene families specific to *Bovidae*, and are derived from the transposition of an autosomal gene block. The temporal and spatial expression patterns of *ZNF280BY*s in testis suggest a role in spermatogenesis. This study offers insights into the genomic organization of the bovine MSY and gene regulation in spermatogenesis, and provides a model for studying evolution of multi-copy gene families in mammals.

## Background

Sex-determination in Eutheria depends on the X and Y chromosomes (X/Y-chr) that evolved from a pair of autosomes less than 166 million years ago (Mya), after the divergence of the monotreme lineage [1,2]. Acquisition of a sex-determining locus (*SRY*) transformed the ancestral autosomes to the proto-X/Y [3] and a subsequent stepwise suppression of meiotic recombination between X and Y led to a male-specific region on the Y-chr (MSY) [3,4]. The remaining euchromatic region of the Y-chr, the pseudoautosomal region (PAR), retains the ability to recombine with the X-chr during meiosis and shares the same genes and DNA sequences with its X counterpart [3,5-9]. The Y-chr, compared to the gene-rich (~1600 genes) and highly conserved X-chr, has degenerated and lost more than 95% of ancestral genes, and is poorly conserved among mammalian lineages [7,9-11]. After recombination was suppressed with the X-chr, degeneration of the Y-chr was driven by several synergistic evolutionary forces, including Muller's ratchet, background selection, the Hill Robertson effect with weak selection and hitchhiking of deleterious alleles by favorable mutations [12,13]. Independent Y-chr decay during evolution [3,14] led to different eutherian lineages retaining different subsets of Y genes, and a diverse and lineage-specific Y-chr gene content.

Male-benefit genes have accumulated on the Y-chr through persistence of genes derived from the proto-X/Y [3], transposition and retroposition from autosomes and subsequent amplification [15-18]. Y-chr gene amplification with an attendant higher level of expression has been suggested to enhance gene function particularly beneficial to the male [4,19]. As a result, gene accumulation and amplification have provided the Y-chr with a functional coherence in sex determination, spermatogenesis and fertility not observed in other regions of eutherian genomes [15], with genes in MSY showing remarkably uniform expression patterns either exclusively or predominantly in the testis [4,19].

To date, several lineage-specific Y-chr gene families, including the human *DAZ, CDY*, the mouse *Ssty1 *and the cat *TETY1 *and *FLJ36031*, have been reported [4,18,20-22]. *DAZ *and *CDY *appear as main candidates for the human *Azoospermia Factor *(*AZF*) [23-32]. Although these two primate lineage-specific Y genes are autosomal in cattle and other non-primates, their functions in spermatogenesis and male fertility are highly conserved in the non-primate autosomal orthologs [4,24,32-34].

Causal reasons for the species-specific accumulation of Y-genes remain elusive, as little information is available regarding the gene content of the Y-chr in most eutherian mammals. We believed that the identification of species-specific Y-genes in individual species will increase our understanding of the mechanisms underlying gene acquisition and evolution, and offer insights into the genes central to regulation of male fertility and spermatogenesis.

The bovine (*Bos taurus*) Y-chr (BTAY) was estimated to be ~1.77% (~51 Mb) of the entire bovine genome (2.87 Gb) [35,36]. The MSY region comprises ~95% of BTAY, approximately 50% of which belongs to the euchromatic region (~24 Mb) [37]. Previous studies on BTAY were based on a comparative mapping approach [37], resulting in several Y-linked genes, such as *AMELY*, *DDX3Y*, *SRY*, *TSPY, UTY *and *ZFY *[38-41]. However, due to the limitation of the comparative mapping approach, lineage-specific Y-chr gene(s) has not been extensively analyzed in bovid.

The purpose of the present study is to investigate the MSY region, and to identify and characterize the bovid lineage-specific Y-chr gene(s). By using a direct testis cDNA selection approach [20,42], we discovered two bovine-specific Y-chr gene families--*ZNF280BY *(*Zinc finger protein 280B Y-link*, also known as *SUHW2*, *suppressor of hairy wing homolog 2*) and *ZNF280AY *(*Zinc finger protein 280A Y-link*, or *SUHW1)*, which originated from a segmental duplication of their paralogs on the bovine chr 17 (BTA17) and were subsequently amplified to over 100 copies on BTAY. To our knowledge, this is the first report on an autosome-to-Y "gene block" transposition. The transposition and amplification of *ZNF280B/ZNF280A *on the bovid Y-chr provides a fundamental model for further studying the expansion of multigene families on Y-chr and for understanding the evolutionary force that shapes the gene functions.

## Results

### Identification of ZNF280BY and ZNF280AY gene families

**Figure 1:**
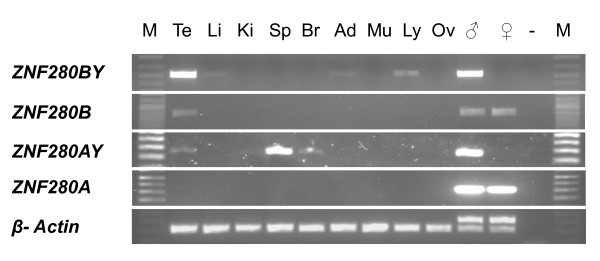
**Tissue expression profiles of *ZNF280B/ZNF280BY *and *ZNF280A/ZNF280AY***. Both *ZNF280BY *and *ZNF280AY *were proved to be Y-specific genes via the male-specific PCR (lane marked with ♂). *ZNF280BY *is expressed predominantly in the testes and slightly in liver, adrenal gland and lymph node while the autosomal *ZNF280B *is expressed specifically in the testis. The *ZNF280AY *is expressed predominantly in the spleen and low in testis and brain, while the expression of the *ZNF280A *was not detected among the eight tissues studied. The *β-actin *gene was used as positive control. Te, testis; Li, liver; Ki, kidney; Spl, spleen; Br, brain (cerebrum); Ad, adrenal gland; Mu, muscle; Ly, lymph node; Ov, ovary; ♂, bovine male genomic DNA control; ♀, bovine female genomic DNA control; -, water; M, 1 kb DNA ladder.

BTAY-expressed sequences were enriched by hybridizing a mixed testis cDNA with a micro-dissected, PCR amplified, biotin-labeled BTAY probe [39,43]. The enriched BTAY cDNAs were sequenced by two different technologies (see Methods). Among the 273 transcripts obtained from the Sanger sequencing, 46 (~17%) matched to an unmapped bovine *ZNF280B *mRNA (NM_001078120.1) with a sequence similarity ranging from 97% to 100%. This mRNA on the Y-chr has differentiated and shares 93% sequence identity with *ZNF280B *on BTA17 (NM_001077935.1). Assembly of ~13 million short (36 bp) reads from the next generation sequencing resulted in ~4,500 sequence contigs (to be published separately). One of the contigs is a full-length cDNA (1,989 bp, GenBank acc. no. HQ014563) with 99% similarity to NM_001078120. Another 3,386 bp contig (GenBank acc. no. HQ014564) is a paralog of the predicted bovine *ZNF280A *(XM_596386.4 and XM_002694686.1) with a sequence similarity of 91%. The bovine *ZNF280B *(aliases: *SUHW2, 5'OY11.1*) (NM_001077935.1) and *ZNF280A *(aliases: *SUHW1, 3'OY11.1*) (XM_596386.4) genes map next to each other on the distal long arm (74.307-74.326 Mb) of BTA17 (Build 4, http://www.ncbi.nlm.nih.gov/gene/517697). We have confirmed, by male-specific PCR, that the NM_001078120 mRNA and the two cDNA contigs paralogous to either *ZNF280B *or *ZNF280A *are all Y-linked in cattle (Figure 1). Therefore, we referred these two genes as *ZNF280BY *or *ZNF280AY.*

In the absence of a BTAY sequence assembly, we blast-searched *ZNF280BY *(NM_001078120) against all sequenced bovine bacterial artificial chromosomes (BACs) deposited in GenBank (HTGS database) and identified 252 BACs (240 annotated Y-BACs, the remaining BACs are draft sequences) that contains a total of 377 copies of *ZNF280BY *(1-5 copies/BAC). A similar search with *ZNF280AY *(acc. no. HQ014564) resulted in the identification of 132 BACs (124 annotated Y-BACs, 8 draft sequence BACs), which all contained the *ZNF280BY *genes. All *ZNF280AY-*BACs harbor a single copy, except for one BAC that contains two separate copies of *ZNF280AY*, resulting in a total of 133 copies of *ZNF280AY*. These results suggest that both *ZNF280BY *and *ZNF280AY *are multi-copy gene families on BTAY.

**Figure 2:**
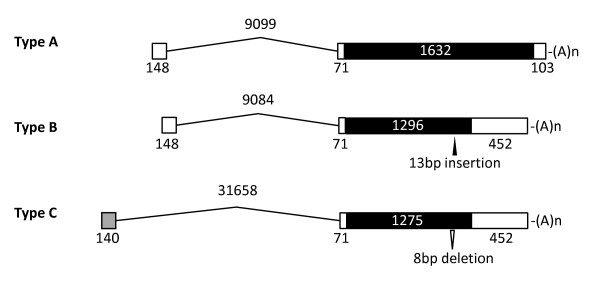
**Genome structures of the bovine *ZNF280BY***. Schematic representation of the three major genomic structures of *ZNF280BY*, denoted as type A, B and C. Type A is the normal form with an estimated 113 copies on BTAY. Type B is resulted from a 13 bp insertion while Type C resulted from an 8 bp deletion in the coding region. The coding segments (CDS) are shaded in black. The number denotes the length of exons, introns and CDS in bp. The non-homologous exon 1 of *ZNF280BY *type C is shaded in grey. The polyA [(A)_n_] sites are indicated.

Point mutations and insertions/deletions (indels) were found to be present in many of the *ZNF280BY *copies based on sequence alignment and open reading frame (ORF) analyses, leading to 241 copies with short or no ORFs. Thus, they are pseudogenes. The remaining 136 copies, in which their ORF varied from 222 aa to 543 aa, were predicted to be active at the transcription level using Splign program [44] (Additional file 1). Among all potential active *ZNF280BY *loci, 113 contain the normal full-length mRNA of 1,954 bp (acc. no. GU144303, Figure 2, type A), and encode a peptide of 543 aa, which is 87% identical to the ZNF280B protein on BTA17 (NM_001077935, 545 aa). The remaining 23 loci encode peptides of 222 to 469 aa, resulted from nonsense mutations or indels (Additional file 1). Two of the short peptides, 431 aa (type B, acc. no. GU144304) and 424 aa (type C, acc. no. GU144305), were investigated in details (Figure 2). All *ZNF280BYs *have two exons with the coding segment (CDS) in exon 2. The only intron in the *ZNF280BY *family is located in the 5'-UTR region, 71 bp upstream of the start codon, and varies in size ranging from 9.1 to 31.7 kb among type A, B, and C. The first exon of type C does not share any homology with type A or B (Figure 2). RACE (rapid-amplification of cDNA ends) and RT-PCR analyses demonstrated that the type B contains a 13 bp insertion at nt 1247 within the CDS, leading to a frame shift and a stop codon (TAA) at nt 1293, and hence, a shorter peptide of 431 aa (Figure 2). Type C has an 8 bp deletion at nt 1218, resulting in a premature stop codon (TAA) at nt 1273 and a 424 aa peptide. In type B and C, only three and two zinc finger motifs were detected, respectively, while the normal ZNF280BY (type A) has four zinc finger motifs.

**Figure 3:**
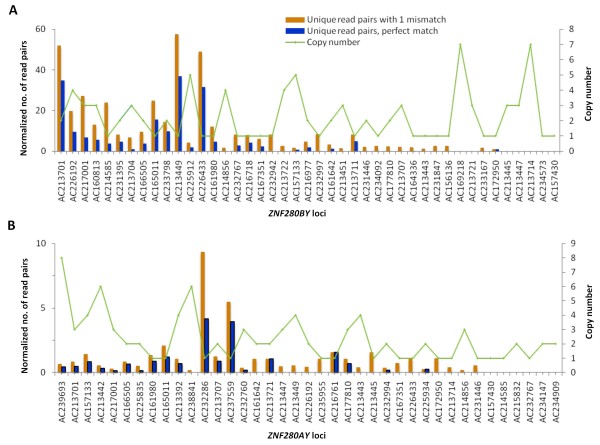
**Locus-specific expression of *ZNF280BY *and *ZNF280AY *in testis**. The alignment of read pairs derived from the deep sequencing of the selected cDNAs against unique coding regions of the *ZNF280BY *(A) and *ZNF280AY *(B) gene families. The left side Y-axis indicates the normalized number of read pairs, and the right side Y-axis indicates the identical copy number for a particular gene locus on BTAY. The blue columns indicate the perfectly matched read-pairs, while the yellow columns show the matched read-pairs with one nucleotide mismatch. The deep sequencing reads counting results are detailed in Additional file 3.

A series of analyses were carried out to verify whether the predicted active loci of *ZNF280BY *are transcriptionally active. First, pairwise alignment of the *ZNF280BY *cDNA sequences obtained from the Sanger sequencing with the Y-BAC genomic sequences identified 28 unique cDNAs that match perfectly to one or more type A sequences (a total of 80), and two additional unique cDNAs matched to type B and C, indicating at least 82 loci of *ZNF280BY *are expressed in bovine testes (Additional file 2). Second, alignment of the *ZNF280BY-*matched Illumina reads (pair-end, 2 × 36 bp) to each of the predicted *ZNF280BY *loci revealed that at least 96 loci are transcriptionally active (Figure 3A, Additional file 3). Since the results from Sanger sequencing and Illumina sequencing overlap ~80%, they complement each other. Third, 5'- and 3'-RACE confirmed the expression of the *ZNF280BY *type B and C. Finally, multiple alignment of promoter sequences indicated that *ZNF280BY *type A promoters are highly conserved (>98% similarity). These results collectively suggest that all predicted *ZNF280BY *loci on BTAY may be transcriptionally active.

For the bovine *ZNF280AY *gene family, the *ZNF280AY *cDNA (acc. no. HQ014564) contains a short ORF (113 aa). Further analysis of all 133 *ZNF280AY *genomic sequences predicted short ORFs ranging from 100 to 262 aa, which are less than half the length of the protein encoded by *ZNF280A *(538 aa). Therefore, we speculate that all *ZNF280AY *loci on BTAY were pseudogenized. However, our Illumina sequence analysis indicated that as many as 91 *ZNF280AY *loci (with an average length of 2620 bp, ranging from 1656 bp to 3386 bp) could be transcriptionally active, and that 51 of them were unquestionably expressed as they matched 100% with unique read pairs (Figure 3B, Additional file 3).

### Distribution of ZNF280BY and ZNF280AY on BTAY

**Figure 4:**
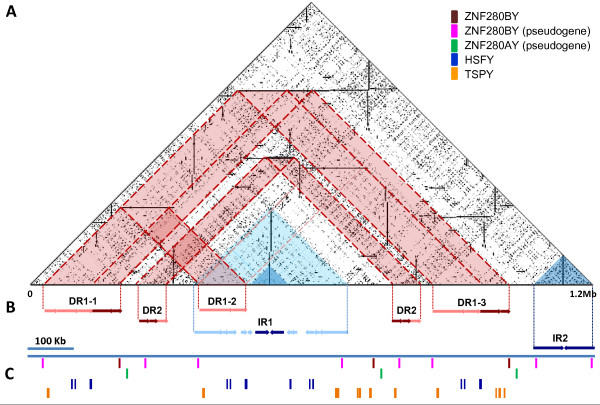
**Sequence analysis of a 1.2 Mb ampliconic region of BTAY**. This region was assembled from nine Y-BACs (see Additional file 4). **A**. Triangular dot-plot analysis of the 1.2 Mb BTAY sequence. Each dot represents a perfect match in a word size of 10 nucleotides [81]. The plot indicates that the BTAY ampliconic region is enriched with internally repetitive units. **B**. Two direct repeats (DR1 and DR2) and two inverted repeats (IR1 and IR2) were detected in the region. The regions with high arm-to-arm similarity are highlighted in red and blue for DR and IR, respectively. The corresponding repeat regions are also highlighted in the dot-plot. The dark red regions highlight the DRs with similarity over 99.5%; the dark blue regions highlight the IRs with similarity over 99.5%. The light red regions are extended direct repeats with similarity over 99% and the light blue regions are extended inverted repeats with similarity over 98.5%. **C**. The predicted transcribed regions of *ZNF280BY*, *ZNF280AY*, *HSFY *and *TSPY *are depicted in different tracks with different colors as indicated in the up-right corner. Scale: bar = 100 kb.

The distribution/duplication patterns of the *ZNF280BY *and *ZNF280AY *loci on BTAY were identified by assembling a contig using the *ZNF280BY*/*AY*-containing BACs deposited in GenBank. These BACs were assembled under stringent criteria that assured overlaps between BACs were ≥30 kb in size with ≥99.99% sequence identity. We obtained a 1.2 Mb contig of nine BACs using Sequencher 4.8 (Genecodes, Ann Arbor, MI) (Figure 4, Additional file 4) that contained 11 loci of *ZNF280BY *(Figure 4), of which three were potentially coding and shared the same structure as *ZNF280BY *type A, while the remainder were pseudogenes. Interestingly, each active *ZNF280BY *is accompanied by a pseudo *ZNF280AY*, forming a *ZNF280BY-ZNF280AY *block with a 19 kb interval. The *HSFY *(*heat shock transcription factor, Y-linked*) and *TSPY *(*testis-specific protein, Y-linked*) gene family also reside within this contig and amplified similarly to *ZNF280BY *(Figure 4). The 1.2 Mb contig may represent a typical ampliconic region reflecting the redundant nature of BTAY DNA.

Interspersed repeats comprise ~58% of the 1.2 Mb contig and contain a high density of long interspersed repetitive elements (LINEs) and retrotransposable elements (RTEs), but low density of short interspersed repetitive elements (SINEs), thus consistent with a previous report on the bovine genome [35]. Two major directed repeats (DRs) are present in this region: DR1 and DR2 (Figure 4). DR1 contains three 110 kb repeat units (DR1-1, -2, and -3), which are 99.40% similar without considering gaps. DR1 has one *ZNF280BY *pseudogene in each repetitive unit. DR1-1 and DR1-3 share an extra 65 kb extended repeat with 99.70% similarity that contains one active *ZNF280BY*. DR2 contains two 64 kb units which are 99.50% similar, as well as pseudogene copies of *ZNF280BY*. There are also two inverted repeats (IRs), IR1 and IR2, over 99.50% similar (Figure 4), which span 60 kb (30 kb per arm) and 134 kb (67 kb per arm), respectively. One pseudogenized *ZNF280BY *was detected in each arm of IR2, but no *ZNF280BY *sequence was present in IR1. However, when the identity threshold of IR1 was lowered to 98.50%, we were able to expand the length of IR1 to 500 kb and identify a *ZNF280BY *pseudogene in each arm. The 1.2 Mb contig apparently contains multiple internally repeat units, with *ZNF280BY, ZNF280AY, HSFY *and *TSPY *representing the major gene families (Figure 4).

### The origin of the ZNF280BY-ZNF280AY block

**Figure 5:**
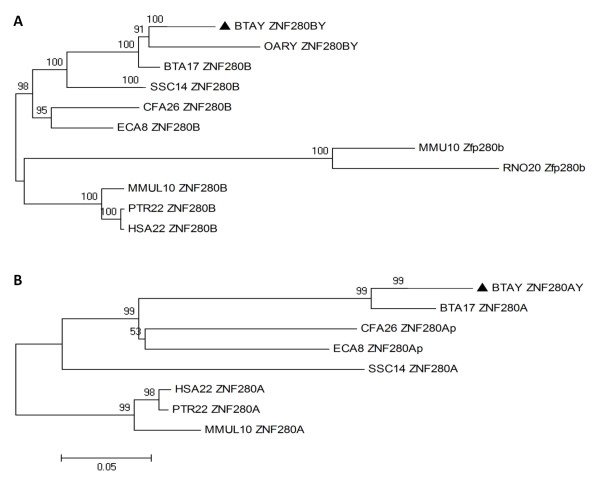
**Phylogenetic trees of *ZNF280B/ZNF280BY *and *ZNF280A/ZNF280AY *gene families**. **A**. *ZNF280B/ZNF280BY *tree. **B**. *ZNF280A/ZNF280AY *tree. The bovine *ZNF280BY *and *ZNF280AY *gene families are consistently clustered with their autosomal paralog on chromosome 17, supporting that these two gene families originated from the transposition of the autosomal gene block. The trees were built based on the Neighboring-joining method implemented in MEGA4 software [77]. The trees are drawn to scale and the evolutionary distances were computed using the Maximum Composite Likelihood method. The bootstrap values (1000 replicates) are shown next to the branches [83]. The filled triangle indicates the condensed branch for each of the gene families. Scale: bar = 0.05 unit of the number of base substitutions per site. HSA = Human; PTR = Chimpanzee; MMUL = Macaque; MMU = Mouse; RNO = Rat; CFA = Dog; ECA = Horse; SSC = Pig; ORA = Sheep; BTA = Cattle.

In order to gain insight into the evolution of the *ZNF280BY *and *ZNF280AY *gene families, we retrieved *ZNF280B/BY *and *ZNF280A/AY *orthologous sequences from 10 mammalian species available to date (Figure 5, Additional file 5) and conducted phylogenetic analysis. We found that the autosomal *ZNF280B*-*ZNF280A *gene block on BTA17 is highly conserved among all sequenced eutherians (except for mouse, rat and elephant), including human, chimpanzee, macaque, cow, dog, pig, and guinea pigs, and even in non-placental vertebrates (opossum, chicken, frog, and zebrafish) (Additional file 6). However, *ZNF280BY*/*AY *orthologs were identified only in bovid (cattle and sheep) Y-chrs. The *Ovis aries Y chromosome repeat region OY11.1 DNA sequence *(acc. no. U30307.1) was first deposited in GenBank in 1995, and confirmed by fluorescent *in situ *hybridization (FISH) to be conserved throughout *Bovidae *[45].

The *OY11.1 *repeat sequence covers the *ZNF280B *(*5'OY11.1*), but not the *ZNF280A *(*3'OY11.1*) (http://www.ncbi.nlm.nih.gov/sites/gene) sequence. The similarity between the ovine and bovine *ZNF280BY *is 88% at the nucleotide level and 86% at the protein level. Due to the unfinished ovine genome sequencing, we were not able to retrieve the ovine *ZNF280B*, *ZNF280A *and *ZNF280AY *sequences in this study.

We built phylogenetic trees (Figure 5) for both gene families using neighbor-joining (NJ) and maximum-likelihood (ML) methods. The topologies were consistent between the NJ and ML tree. All predicted *ZNF280BY *(type A, B, and C) cDNAs (Additional files 1 and 2) were grouped in a single cluster (Figure 5A). The bovine *ZNF280BY *gene family together with the ovine *ZNF280BY *(*OY11.1*) were clustered with the bovine BTA17 *ZNF280B *and formed one clade with a perfect bootstrap support (Figure 5A). The autosomal orthologs of the bovine *ZNF280B *in eight mammalian species were clustered in a different clade, also with a strong bootstrap support (95-100%) (Figure 5A). The phylogenetic tree of the bovine *ZNF280A/ZNF280AY *(Figure 5B) is very similar to the one of *ZNF280B/ZNF280BY*, although mouse and rat were excluded in the analysis as their orthologous sequences were not available. The facts that the *ZNF280B/ZNF280A *gene block is located in a region of conserved synteny in eutherians and that the *ZNF280BY *and *ZNF280AY *gene families are present in bovid only, strongly suggest that the Y-linked families were a transposition of the autosomal (BTA17) block. This autosome-to-Y transposition event occurred most likely before the divergence of cattle and sheep, which was estimated to be ~19.6 Mya [46].

We estimated the age of the active *ZNF280BY *loci by using the bovine and ovine *ZNF280BY *sequences with the neutral rate estimates as the molecular clock (Figure 5A). The mean Ks value between bovine and ovine *ZNF280BY *is 0.1517. The maximum pairwise Ks value of the most distant *ZNF280BY *locus (AC172950) to the remaining active loci in bovine is 0.0443. Based on the previous estimation of the divergence time of 19.6 Mya between the two species [46], we estimated that the duplication events for the current active *ZNF280BY *loci occurred approximately 5.7 Mya (0.0443/0.1517 × 19.6) [47].

### The expression profile of ZNF280BY and ZNF280AY

RT-PCR analyses revealed that *ZNF280BY *expression is predominant in testis but low in liver, adrenal gland and lymph node, while the autosomal *ZNF280B *expression is specific to testis (Figure 1). The expression of *ZNF280AY *pseudogenes is high in spleen but low in testis and brain, while the *ZNF280A *expression was not detected among the nine tissues tested (Figure 1).

**Figure 6:**
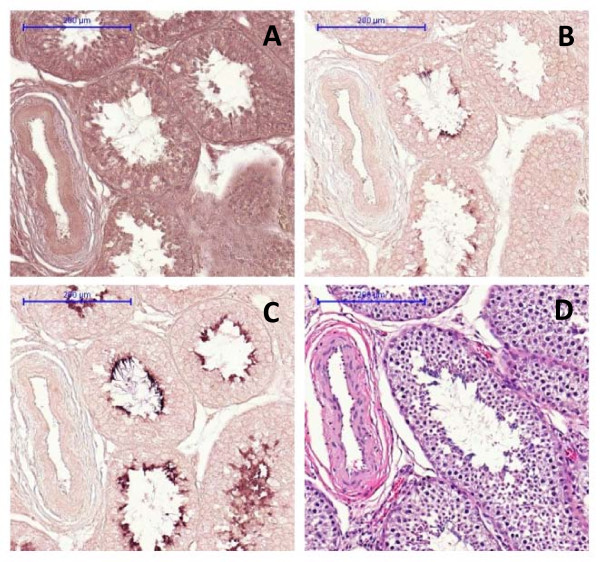
**Tissue localization of the bovine *ZNF280BY *transcript in adult testis**. The sense and antisense RNA of *ZNF280BY *are expressed in adult testis. **A**. The *ZNF280BY *sense RNA is expressed widely and evenly across all cell types in the seminiferous tubules. **B**. The antisense RNA of *ZNF280BY *was only detected in spermatids. Sense (A) and antisense (B) RNA of *ZNF280BY *were detected by the corresponding DIG-labeled cRNA probes. **C**. The bovine *Protamine *gene was used as the positive control, and there is no antisense mRNA of *Protamine *detected in the bovine testis [34]. **D**. The Haematoxylin and Eosin (H&E) staining was shown. Scale: bar = 200 μm.

*In situ *hybridization (ISH) with *ZNF280BY *indicated that the sense and antisense RNA of this gene are expressed in adult testis (Figure 6). *ZNF280BY *sense RNA was widely expressed (Figure 6A), but the antisense RNA was detected only in the spermatids (Figure 6B).

**Figure 7:**
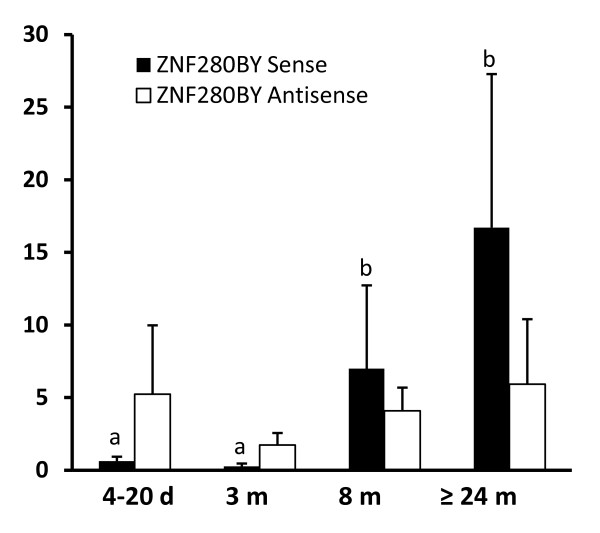
**Expression of *ZNF280BY *sense and antisense RNA at different developmental stages of testis**. The relative expression levels of the *ZNF280BY *sense and antisense RNA at different ages of testis (X-axis), measured by the strand-specific qPCR, were normalized by the 18S rRNA (Y-axis). Values are means ± SD of triplicates. The expression of sense RNA increased gradually with age. In contrast, the *ZNF280BY *antisense RNA had a stable expression level except for a decrease in 3 month old testis. The significant expression difference was identified when p ≤ 0.05 by the ANOVA analysis and denoted by (a,b) where the gene expression at stages of (b) is significantly different from that at (a).

The expression of the sense and antisense RNA of the *ZNF280BY *over different stages of testis development (4~20-day, 3-month, 8-month and >24-month) by qRT-PCR indicated the expression of *ZNF280BY *sense RNA increased significantly with age (p < 0.05). In contrast, *ZNF280BY *antisense RNA expression was stable during testis development (Figure 7). It is noteworthy that expression between qRT-PCR replicates for the same individual at each stage (technical replicates) were highly consistent, whereas the levels between different individuals (3 biological replicates) varied, resulting in relatively high standard deviations (Figure 7).

To address the question of whether different loci of the *ZNF280BY *families on BTAY are differentially expressed, we counted the number of read-pairs unique to a given locus (read-pairs that are shared by two or more loci were not counted). As shown in Figure 3A, different loci of *ZNF280BY *are differentially expressed with as much as 60-fold changes. Similar analysis to the different copies of the *ZNF280AY *pseudogenes on BTAY indicated that their expression also varied as much as 10-fold (Figure 3B). However, the expression level of the *ZNF280AY *pseudogenes is much lower (one-sixth) compared to the *ZNF280BY *genes (Figure 3), which is consistent with the RT-PCR analysis (Figure 1).

## Discussion

### Autosome to Y-chr transposition

Autosome-to-Y transposition of male fertility genes is a recurrent theme in mammalian Y-chr evolution [48]. As a result, the content of male-beneficial genes in MSY has increased in spite of a 95% loss of the ancestral Y-chr genes due to the absence of recombination. Autosome-to-Y transposition events apparently occurred separately in different lineages with newly acquired Y-chr genes from diverse genomic locations [20]. This resulted in lineage-specific Y-chr genes (families) that account for a significant portion of the gene (and sequence) variation among mammalian Y-chrs. The human *DAZ *gene family was derived from the transposition of the autosomal *DAZL *that maps to the sub-telomeric region on HSA3p24.3 [48], while *CDY *arrived on the human Y-chr through retrotransposition of *CDYL *on HSA6 [4,49] during primate evolution. The mouse *Ssty1 *was derived from a retroposition of an autosomal gene *Spin1 *on MMU13 [18]. The feline *FLJ36031*and *TETY1 *gene families originated through autosome-to-Y transposition before and after the divergence of cats and dogs, respectively [20].

In addition to autosome-to-Y transpositions, a massive X-to-Y sequence transposition has also been observed in humans, which occurred after the human and chimpanzee divergence (~3-4 Mya) and resulted in an X-transposed sequence block on the Y that contains two single-copy genes, *TGIF2LY *and *PCDH11Y *[4]. This block of the Y-chr sequence still shares a high similarity with the X-counterpart (~99%) though it does not recombine during meiosis [4].

We identified two novel Y-chr gene families, *ZNF280BY *and *ZNF280AY*, which are present on both bovine and ovine Y-chrs, but not on non-bovid Y-chrs investigated to date. We propose that the *ZNF280BY/AY *gene families are lineage-specific in *Bovidae*. Phylogenetic analysis of the *ZNF280B/BY and ZNF280A/AY *family (Figure 5) strongly support that the bovine *ZNF280BY/AY *originated from the autosomal genes, *ZNF280B/A*, on BTA17. Unlike the human DAZ and feline *TETY1 *and *FLJ36031 *genes, where a single autosomal gene is involved in the transposition, the bovine *ZNF280B/A *transposed to the Y-chr as a block. This autosomal block is located in the sub-telomeric region that could be a hotspot for segmental duplication [50].

### ZNF280BY-ZNF280AY amplification and the "birth-and-death" evolution

The distribution pattern and differences in copy number suggest that *ZNF280BY-ZNF280AY *was differentially amplified on BTAY after transposition. We identified 136 active *ZNF280BY *and 241 pseudogenes from BTAY-BACs, and estimated that the oldest active *ZNF280BY *locus emerged via a duplication event ~5.7 Mya. This is significantly later than the estimated divergence time of 19.6 Mya between cattle and sheep [47]. The results lead us to believe that the progressive amplification of the bovine *ZNF280BY *follows the "birth-and-death" model of evolution [51]. Based on this model, new *ZNF280BY *loci were created by repeated gene duplication with some duplicated genes maintained on the BTAY and others lost due to mutation and degeneration, as active gene copies might cooperate to increase the efficiency of male fertility and damage to a few copies would not significantly impact the biological system [52]. Each of the three active *ZNF280BY *loci in the 1.2 Mb region is accompanied by a *ZNF280AY *pseudogene (Figure 4). If this is true for all 136 active *ZNF280BY *loci, we should expect to identify a minimum of 136 *ZNF280AY *pseudogenes. Interestingly, the observed 133 copies of *ZNF280AY *pseudogenes in this study are very close to the expected number, but we do not, as yet, know whether co-amplification of the *ZNF280BY-ZNF280AY *or the loss of the *ZNF280AY *in the gene pair has any role in the activity of *ZNF280BY.*

A significant feature of the human MSY is the eight palindromes (24 kb to 2.9 Mb) with arm-to-arm sequence identities ≥99.94% [4,53]. Y-to-Y gene conversion between the paired-genes on each arm occurs frequently in humans, constraining sequence divergence and maintaining the critical functions of the MSY genes [4,53]. Pairwise comparisons of all available BTAY-BACs and the assembly of a 1.2 Mb ampliconic region located in MSY revealed several palindrome-like inverted repeats with an arm-to-arm identity of 99.85% (Figure 4). The occurrence of palindrome-like IRs indicates that the Y-Y gene conversion mechanism may apply to these gene families. When the criteria for sequence assembly was lowered from 99.99% to 99.85% identity, and the minimum overlap size between BACs decreased from 30 kb to 20 kb, we obtained another large contig (~2.2 Mb) from different Y-BACs displaying similar dot-plot and repeat pattern as the 1.2 Mb contig (Figure 4). It appears that the majority of the bovine MSY ampliconic region is enriched with internally repetitive units containing *ZNF280BY*, *ZNF280AY*, *HSFY *and *TSPY*.

### Locus-specific expression of multi-copy genes on the Y-chr

One of the most important features of mammalian Y-chrs is the multi-copy gene families that are exclusively expressed in testis and play essential roles in spermatogenesis and male fertility. The largest Y-gene family reported to date is *TSPY*, which is conserved on most of the mammalian Y-chrs. The human Y-chr harbors 20-70 copies of *TSPY *[4,54], while the bovine Y-chr has 50-200 copies [55-57]. Unlike the human *TSPYs *that are clustered as a tandem array in a narrow region (~700 kb) on Yp [4], the bovine *TSPYs *spread over the majority of the MSY ampliconic region. Recent studies in *TSPY *copy number variation (CNV) has indicated that CNV significantly influenced spermatogenic efficiency in men [58,59]. However, efforts to study the expression profile of the multi-copy Y-chr genes have been impeded because of the high similarity (95-100%) among different copies (loci). In this work, we have successfully applied the deep sequencing technology to analyze the locus-specific expression of the *ZNF280BY *and *ZNF280AY *gene families. We believe that there are two important advantages with the deep sequencing approach. First, by counting the perfectly matched read pairs, one can directly evaluate the expression level of any given locus (except for those loci that are 100% identical). Second, one can also compare the average expression level among gene families or individuals (biological replicates) based on read counts. We expect that the combination of CNV and locus-specific expression analyses on these Y-gene families, including *ZNF280BY*, *ZNF280AY*, *HSFY*, and *TSPY*, will help us to understand how multi-copy gene families regulate and function during spermatogenesis.

### Potential function in spermatogenesis

Although zinc finger proteins are among the most abundant and functionally diverse in mammalian genomes [60], little is known of the functions of *ZNF280B*(*SUHW2*) in mammals. The Drosophila ortholog, *suppressor of Hairy-wing *[*Su(Hw)*], is a transcriptional regulator. The Su(Hw) protein has been found to bind an insulator element within a gypsy retrotransposon to mediate enhancer-blocking of gypsy towards target genes [61,62]. The conserved syntenic block *ZNF280B*-*ZNF280A *on HSA22 is located within the lambda light chain loci and is reportedly associated with immune responsiveness [63,64].

The discovery of the autosome-to-Y transposition and subsequent amplification of the *ZNF280BY-ZNF280AY *gene block on BTAY led us to suggest that the two gene families may play an essential role in spermatogenesis. A comparable example is the *Ssty1 *gene family in mice which is significantly amplified (>100 copies) after retroposing to the Y-chr and is expressed specifically in spermatids with a function in sperm differentiation [65,66]. As expected, we found that the bovine *ZNF280BY *family predominantly and *ZNF280B *specifically expressed in testes (Figure 1). The significant age related elevation of *ZNF280BY *levels (Figure 7) may be associated with the maturation of the bovine testes and cell proliferation throughout spermatogenesis. Antisense RNA may play a role in regulating the expression of *ZNF280BY *during spermatogenesis (Figure 6A &6B). The antisense transcripts of *ZNF280BY *were over-expressed relative to sense transcripts in testes ≤3 months of age (Figure 7). This observation is in contrast to a previous report that found lower antisense versus sense transcript levels in genes expressed in germ cells [67]. The antisense RNAs of the three Y-related and testis-expressed genes in cattle, including *ZNF280BY*, *DDX3Y *and *CDYL*, all appear to be expressed in the late stage of spermatocytes and/or spermatids, indicating that antisense RNA is important in regulating bovine spermatogenesis [34,68].

## Conclusions

The identification of lineage-specific Y genes recapitulates the diversity of Y gene content and signifies the importance of comparative studies of the mammalian Y-chr. This study provides a base for further research on Y-chr evolution and male-fertility. As the expression of the bovine *ZNF280B/ZNF280BY *and *ZNF280A/ZNF280AY *is predominant in the testis, their autosomal orthologs in other eutherians may play roles in spermatogenesis. We believe that additional functional analysis of *ZNF280B/ZNF280BY *and *ZNF280A/ZNF280AY *will offer insights into gene regulation in spermatogenesis, the evolution of the mammalian Y-chr and advance the assembly of the bovine Y-chr sequence.

## Methods

### RNA extraction and cDNA synthesis

Total RNA was extracted from bovine testicular tissue at 4 days, 20 days, 3 months, 8 months and ≥2 years of age with Trizol^® ^reagent (Invitrogen, Carlsbad, CA, USA). Equal amounts of total RNA from different age groups were pooled, treated twice (before and after mRNA purification) with the DNase I (Ambion, Austin, Texas, USA) and messenger RNA was purified from the pooled total RNA (Oligotex; Qiagen, Valencia, CA, USA). First strand cDNA was synthesized with random hexamers and oligo-T primers and SuperScript™ III reverse transcriptase (Invitrogen, Carlsbad, CA, USA) and blunt-ended double-stranded cDNA was developed as described [69]. Adaptors, which are annealed phosphorylated oligonucleotides 1 (5’-CTGAGCGGAATTCGTGAGACC-3’) and 2 (5’-CCAGAGTGCTTAAGGCGAGTCAA-3’) (IDT, Coralville, IOWA, USA), were attached to cDNA using T4 polynucleotide kinase (NEB, Ipswich, MA, USA) [42]. Adaptor-ligated cDNA products were used for direct testis cDNA selection.

### Y-chr DNA probe labeling

Fragments of BTAY DNA were isolated by a micro-dissection approach [70], and were labeled with biotin-16-dUTP (Roche, Indianapolis, IN, USA) using nick translation (Roche, Indianapolis, IN, USA) following a method described by Del Mastro and Lovett [42]. A reaction mixture containing DNA polymerase I, DNase I, 60 ng whole chromosome amplified (WCA) Y fragments, biotin-16-dUTP 0.2 nmol, dNTPs 4 nmol, ^32^P-dCTP (Perkin Elmer, Waltham, Massachusetts, USA) 10 μCi, was incubated in 15°C water bath for 90 min. The biotinylated products were purified through a Probquant G-50 spin column (GE Healthcare, Buckinghamshire, UK) and labeling efficiency was determined by incorporation of ^32^P-dCTP as described [42].

### Direct testis cDNA selection and sequencing

Direct testis cDNA selection was carried out as described [42]. After a pre-hybridization step with bovine Cot-1 DNA (Applied Genetics Laboratories, Melbourne, FL, USA) to block the repetitive elements, the adaptor-ligated cDNA was hybridized with the biotinylated BTAY probe for 50 hr in 0.75 mM NaCl, 20 mM sodium phosphate (pH 7.2), 5 mM EDTA, 5× Denhardt's solution and 0.1% SDS. Hybridized cDNA was isolated with streptavidin paramagnetic Dynabeads M-280 (Invitrogen, Carlsbad, CA, USA) per the manufacturer's instructions. After washing twice with wash solution I (1 × SSC, 0.1% SDS) for 15 min at room temperature to remove the unbound cDNAs, and washing with wash solution II (0.1 × SSC, 0.1% SDS) 4 times, 20 min each at 65°C to remove the non-specifically bound cDNAs, the selected cDNA was eluted from the beads by 1 × SSC at 95°C for 5 min, and then amplified by PCR with the adaptor oligo 1 as the primer. Selection efficiency was assessed by qPCR with Y-linked genes, *SRY *and *DDX3Y*, as positive controls and, *β-Actin *and *CDYL*, as negative controls. PCR products were cloned using a TOPO-TA cloning kit (Invitrogen, Carlsbad, CA, USA). A total of 2,208 random clones were grown overnight at 37°C in 2 ml, 96-deep-well culture plates. Dot-blotting the clones with BTAY fragments yielded 753 (most likely) non-redundant clones. Plasmid DNA was purified by alkaline lysis (Qiagen, Valencia, CA, USA), and sequenced on an ABI-3730XL DNA analyzer at the Pennsylvania State University Genomics Core Facility.

### RT-PCR

Total RNA was extracted from eight different tissues (including testis, liver, kidney, spleen, cerebellum, adrenal gland, longissimus muscle, and lymph node) of a two years old bull and ovarian tissue from a mature cow, treated with DNase I (Ambion, Austin, TX, USA) and reverse transcribed using SuperScript™ III First-Strand Synthesis System (Invitrogen, Carlsbad, CA, USA). RT-PCR was performed with gene-specific primers (GSPs) (Additional file 7) in a 20 μl volume containing 10 ng cDNA, 200 μM dNTPs, 1.5 mM MgCl_2_, 2.5 μM of each primer and 1 unit Taq DNA polymerase (Bioline, Taunton, MA, USA). The PCR conditions were: 94°C for 7 min followed by 35 cycles each of 95°C for 40 sec, 55-65°C for 40 sec, 72°C for 40 sec, with a final extension at 72°C for 7 min. Products were resolved on 1.5% agarose gels with ethidium bromide in 1 × TAE buffer.

### RACE

Total RNAs from bovine testis (4 d, 20 d, 3 m, 8 m, and 2 years of age) were used for 5' and 3' rapid amplification of cDNA ends (RACE). The RACE cDNA template was synthesized using the ExactSTART Eukaryotic mRNA 5'- & 3'-RACE kit (Epicentre, Madison, WI, USA) according to the manufacturer's protocol, and amplified by PCR with kit supplied primers 1 and 2. The 5'-end of *ZNF280BY *was amplified by a nested PCR with Primer 1 and GSPs (Additional file 7). PCR was performed in 20 μl with 10 ng of cDNA (or 1 μl of the first PCR products), 0.25 μM of both primers, 200 μM of each dNTP, 2.25 mM MgCl_2_, 0.6 U of hot-start DNA polymerase (Qiagen, Valencia, CA, USA) under the following cycling conditions: 15 min at 95°C; 35 cycles of 40 sec at 95°C, 40 sec at the annealing temperature (Additional file 7), 1 min at 72°C; and a final extension of 7 min at 72°C. The PCR products were electrophoretically separated in 1.5% agarose, purified with the QIAquick Gel Extraction Kit (Qiagen, Valencia, CA, USA) and sequenced using GSP primers.

### *In situ* hybridization (ISH)

The bovine testis was fixed [71], embedded in paraffin and sectioned (4 μM). Sense and antisense RNA of *ZNF280BY *were selected (Additional file 8) using G-PROBE software (Genetyx Co. Tokyo, Japan). The selected 120 bp probes were subjected to *in vitro *transcription to produce digoxigenin (DIG)-labeled cRNA with the AmpliScribe T7-Flash Transcription Kit (Epicentre, Madison, WI, USA). Uniform labeling of DIG-labeling was confirmed using the NBT/BCIP detection system (Roche Diagnostics, Indianapolis, IN, USA). ISH [72] was modified by hybridizing in 50% formamide, 2 × SSC, 1.0 mg/ml tRNA, 1.0 mg/ml salmon sperm DNA, 1.0 mg/ml BSA, 1.0% SDS and 3.0 μg/ml DIG-labeled RNA probe at 42°C for 26-64 hr. Serial tissue sections were used for antisense and sense probe hybridizations. The spermatid-specific genes *Protamine 1 *(*PRM1*) and *LNE120 *served as positive and negative controls.

### Strand-specific quantitative PCR (qPCR)

First strand sense and antisense cDNAs were synthesized with strand-specific primers (Additional file 7) (SuperScript™ III First-Strand Synthesis System, Invitrogen Carlsbad, CA, USA) using the bovine testis total RNA (4-20 d, 3 m, 8 m and ≥2 years) as templates, which were then used for the real-time qPCR. All qPCRs were performed in Power SYBR Green PCR Master Mix (Applied Biosystems, Foster City, CA) and Applied Biosystems 7500 Real-time PCR system following the manufacturer's instructions. Amplification conditions were 2 min at 50°C; 10 min at 95°C; followed by 40 cycles of 20 sec at 95°C, 20 sec at 57°C and 30 sec at 72°C. Cycle threshold (CT) acquisition used default parameters with CT values for *ZNF280BY *sense/antisense RNAs normalized to 18S rRNA in each sample. RNA samples without reverse transcript served as the negative control. Each qPCR was conducted in duplicate on 3 independent RNA (age) samples (replicates). Significance was evaluated by one-way ANOVA using SAS (SAS Institute Inc., NC, USA).

### Short-read sequencing for locus-specific expression

The selected cDNAs were subjected to mechanical fragmentation by nebulization (compressed air at 32-35 psi for 6 min on ice). All enzymes used for sequencing were obtained from Illumina, Inc. The resulting double-stranded (ds) overhang fragments were end-repaired by incubation in the presence of T4 DNA polymerase and Klenow polymerase. The polished fragments were phosphorylated by T4 polynucleotide kinase, followed by the addition of a single 'A' base to the 3' end of the blunt-ended phosphorylated fragments. This 'A' base prepared the DNA fragments for ligation to adapter oligonucleotides (Illumina paired-read adapters), which have an overhanging 'T' base at their 3' end. Ligation products were size-selected by gel electrophoresis and purification (2% low-range agarose with ethidium bromide). Following 1-2 hr at 80-110 V (room temperature), the library range was visualized under brief UV and the desired size (200-300 bp) was excised. Purified DNA libraries were subjected to a final PCR amplification step (15 cycles). PCR conditions were an initial 30 sec 98°C denaturation, followed by 15 cycles of: 40 sec at 98°C, 30 sec at 65°C, 30 sec at 72°C, followed by 5 min at 72°C and a final hold at 4°C. Amplified libraries were quantitatively and qualitatively assessed by Nanodrop ND-1000 (Thermo Scientific, DE, USA) UV/Vis spectroscopy and DNA BioAnalyzer 2100 microfluidics (Agilent, CA, USA).

A total of 6,710,574 high-quality paired-end reads of 2 × 36 bp were generated using Illumina GAIIx from the selected cDNA. These reads were aligned to the unique *ZNF280BY *and *ZNF280AY *sequences identified through BlastClust with 100% similarity and 100% coverage as the criteria. For aligning the short-reads, the software GSNAP [73] was used as part of the Alpheus pipeline [74]. Two mismatches were allowed during the alignment step and only the reads that hit the reference uniquely were considered for counting towards locus-specific expression. Since the reads were paired-end, only the reads where both ends hit the same reference were considered. These counts were further sub-grouped under three categories: (A) both reads unique hits with 2 mismatches, (B) both reads unique hits with at least one of them being exact match and (C) both reads unique hits & both exact matches. The read counts in these three categories were considered a measure of expression pertaining to the specific locus.

The count values were then normalized by the transcript length ratio and number of unique sites:

$$\frac{\text{Read counts}}{\text{Copy number}}\times \frac{\text{Average length}}{\text{Length}}\times \frac{\text{1}}{\text{Number of unique sites}}$$

### Sequence alignment, gene prediction and phylogenetic tree construction

The *ZNF280BY *(NM_001078120) sequence was Blasted against the annotated Y-BAC pool in NCBI (http://www.ncbi.nlm.nih.gov/) to detect potential homologous regions on BTAY. The SIM4 program [75] was used to compare RACE and RT-PCR results with identified Y homologous regions to determine the homologs with similar intron/exon structures and consensus (GT-AG) splice sites. BlastClust (NCBI package) was used to cluster the retrieved homologs. Open reading frames of these homologs were then predicted with the GETORF program in EMBOSS [76]. Pseudogenes were distinguished from genes on the basis of premature stop codons or frameshifts.

Ks values were calculated using the Nei-Gojobori method (Jukes-Cantor correction) in MEGA4 [77,78]. All positions containing alignment gaps and missing data were eliminated only in pairwise sequence comparisons (Pairwise deletion option). Phylogenetic trees (Additional file 5) were constructed using the NJ and ML methods [77]. The evolutionary distances were computed using the Maximum Composite Likelihood method [79].

### Y-chr sequence assembly

Sequencher 4.8 (Genecodes, Ann Arbor, MI) was used to assemble the BTAY contigs. A cutoff threshold of ≥99.99% sequence identity between overlapped BACs with ≥30 kb overlapped regions ensure a high quality assembly of the 1.2 Mb contig (Figure 4, Additional file 4). The 2.2 Mb contig was assembled under a sequence cutoff threshold set at ≥99.85% identity with ≥20 kb overlapped regions. Repeat elements were identified by RepeatMasker [80]. The dot-plot was produced by Gepard with a word size of 10 nucleotides [81]. Several purpose-designed scripts were coded to visualize the assembly, marker labeling and incorporate the Blast [82] and EMBOSS [76] programs.

## Authors' contributions

YY participated in the design of the study, carried out the cDNA selection, cloning and sequencing, gene structure and expression analyses, and drafted the manuscript. TCC performed the bioinformatics and phylogenetic analysis, Y chromosome sequence assembly, deep-sequencing analysis, and drafted the manuscript. HY carried out the ISH experiment. AKB and EFR performed the deep-sequencing of the selected cDNA and contig assembly. WSL conceived and designed the study, interpreted the results and revised the manuscript critically. All authors read and approved the final manuscript.

## Supplementary Material

Additional file 1 **A list of the active copies of the bovine *ZNF280BY *cDNA sequences and the predicted sizes of ZNF280BYpeptides**. This file provides a list of the active copies of the bovine *ZNF280BY *cDNA sequences, the predicted ZNF280BYpeptides, and the information about the *ZNF280BY *loci were detected in the direct testis cDNA selection. Click here for file

Additional file 2 **Analyses of the *ZNF280BY *containing BACs**. The *ZNF280BY *genomic structure and clusters of predicted cDNAs and protein isoforms. Click here for file

Additional file 3 **Deep sequence reads count for locus-specific expression of the bovine *ZNF280BY *and *ZNF280AY***. Analysis of the locus-specific expression of the bovine *ZNF280BY *and *ZNF280AY *using a deep-sequencing approach. Click here for file

Additional file 4 **BTAY-specific BACs used for assembly of the 1.2 Mb contig**. A list of BTAY-specific BACs used for assembly of the 1.2 Mb contig. Click here for file

Additional file 5 **Sequence information for the phylogenetic trees**. A list of sequences used for phylogenetic analysis. Click here for file

Additional file 6 **The alignment of the *ZNF280B/ZNF280A *gene block across 17 species**. The *ZNF280B/ZNF280A *gene blocks are conserved in the syntenic regions in most mammals except the rodents, where the block was rearranged in two different chromosomes (Chr4/10 in the mouse and Chr5/20 in the rat). This plot was generated based on the human Chr22 assembly (hg18). The boxes represent ungapped alignments; the lines represent gaps. This plot was generated using lastz alignment from the UCSC genome browser (http://genome.ucsc.edu/). Click here for file

Additional file 7 **Primer sequences for (RT-) PCR and strand-specific quantitative RT-PCR**. Primer sequences designed for (RT-) PCR and strand-specific quantitative RT-PCR. Click here for file

Additional file 8 **Probe sequences for the testis section *in situ *hybridization**. *ZNF280BY *sense and antisense cRNA probe sequences designed for the testis section *in situ *hybridization. Probe sequences for the bovine *Protamine 1 *(*PRM1*) gene (positive control) and LNE120 (negative control) are also included. Click here for file
