# Supplementary material for: ZNF280BY and ZNF280AY: autosome derived Y-chromosome gene families in Bovidae
Source: BMC Genomics. 2011 Jan 7;12:13. doi: 10.1186/1471-2164-12-13 (PMC3032696; doi:10.1186/1471-2164-12-13)
Supplement: Additional file 4 — BTAY-specific BACs used for assembly of the 1.2 Mb contig. A list of BTAY-specific BACs used for assembly of the 1.2 Mb contig. [file 1471-2164-12-13-S4.DOCX]

**Additional file 4.** BTAY-specific BACs used for assembly of the 1.2Mb contig.

| Y-BAC name | Accession number |
| --- | --- |
| CH240-242E2 | AC231835.3 |
| CH240-200P13 | AC226022.3 |
| CH240-273J16 | AC232927.2 |
| CH240-168D24 | AC231834.3 |
| CH240-291K12 | AC225839.4 |
| CH240-79P19 | AC213709.3 |
| CH240-237N14 | AC229882.3 |
| CH240-462A20 | AC229799.4 |
| CH240-241F11 | AC233970.3 |
