# Supplementary figures and images for: ZNF280BY and ZNF280AY: autosome derived Y-chromosome gene families in Bovidae
Source: BMC Genomics. 2011 Jan 7;12:13. doi: 10.1186/1471-2164-12-13 (PMC3032696; doi:10.1186/1471-2164-12-13)

## Slide 1
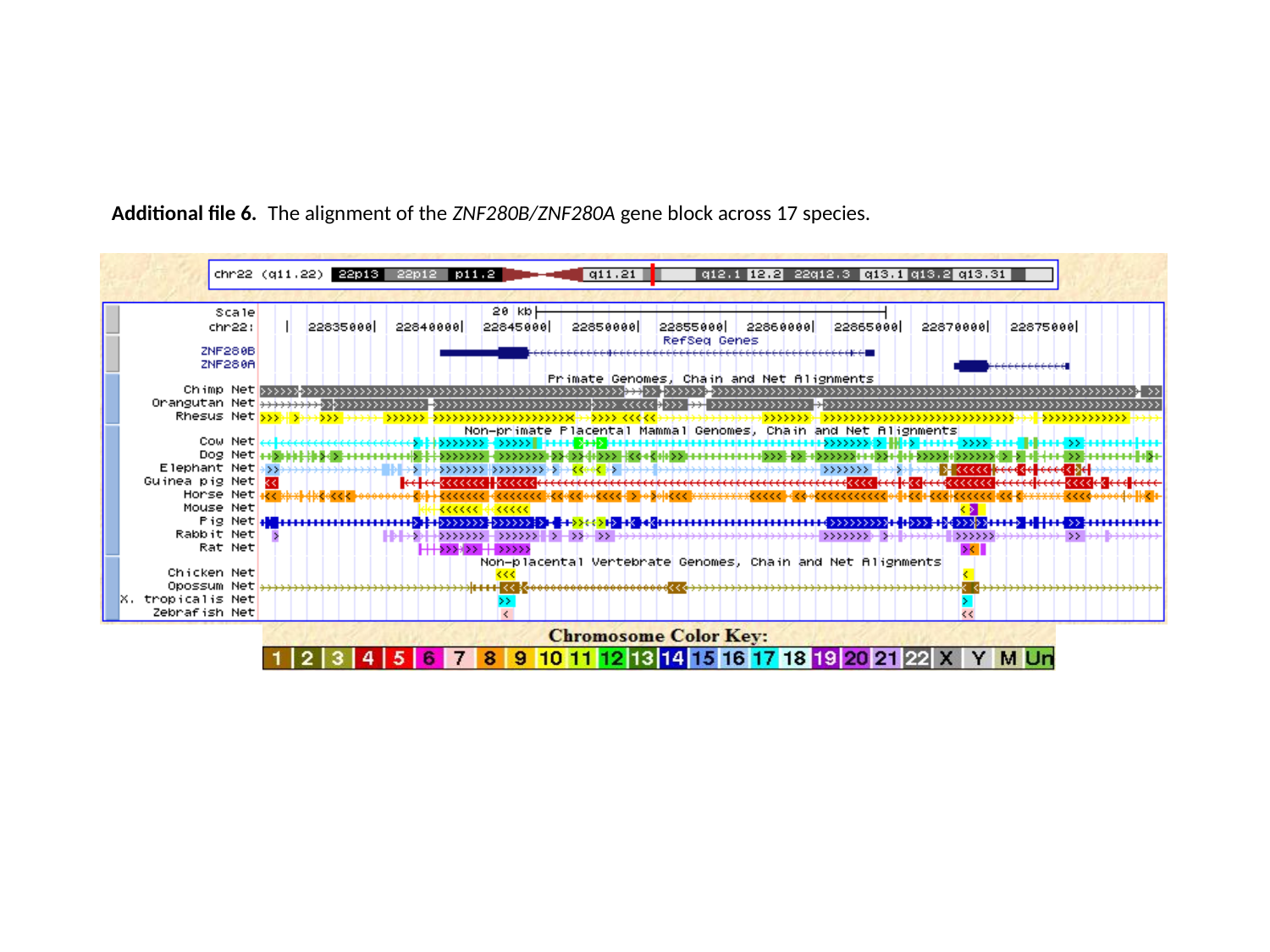

Additional file 6. The alignment of the ZNF280B/ZNF280A gene block across 17 species.

Supplement: Additional file 6 — The alignment of the ZNF280B/ZNF280A gene block across 17 species. The ZNF280B/ZNF280A gene blocks are conserved in the syntenic regions in most mammals except the rodents, where the block was rearranged in two different chromosomes (Chr4/10 in the mouse and Chr5/20 in the rat). This plot was generated based on the human Chr22 assembly (hg18). The boxes represent ungapped alignments; the lines represent gaps. This plot was generated using lastz alignment from the UCSC genome browser (http://genome.ucsc.edu/). [file 1471-2164-12-13-S6.PPTX]
