# Supplementary material for: ZNF280BY and ZNF280AY: autosome derived Y-chromosome gene families in Bovidae
Source: BMC Genomics. 2011 Jan 7;12:13. doi: 10.1186/1471-2164-12-13 (PMC3032696; doi:10.1186/1471-2164-12-13)
Supplement: Additional file 7 — Primer sequences for (RT-) PCR and strand-specific quantitative RT-PCR. Primer sequences designed for (RT-) PCR and strand-specific quantitative RT-PCR. [file 1471-2164-12-13-S7.DOCX]

**Additional file 7.** Primer sequences for (RT-) PCR and strand-specific quantitative RT-PCR.

| Primer name | Sequence | Annealing  temp (℃) | Purpose |
| --- | --- | --- | --- |
| ZNF280BY_5GSP1 | TCACATGACATCCATGGTGG | 60 | Type A, B and C 5' RACE GSP1 |
| ZNF280BYab_5GSP2 | CAGCTCACCTGCACCTAAAC | 60 | Type A and B 5' RACE GSP2 |
| ZNF280BYc_5GSP2 | CATCTTTTCCTCCTGGTTGC | 60 | Type C RACE GSP2 |
| ZNF280BYF | GAACCACCATGGATGTCATG | 57 | RT-PCR forward primer for amplification the middle part |
| ZNF280BYR | TGCTACTTCCACCAATCCTG |  | RT-PCR reverse primer for amplification the middle part |
| ZNF280BYRTF | GTGACTACTCAGCAAAGCAC | 57 | RT-PCR forward primer for expression pattern |
| ZNF280BYRTR | TGTAAGAGAATCTGATCTGC |  | RT-PCR reverse primer for expression pattern |
| ZNF280BY_SRT | CTCCTTGAACAAGAACCTGG |  | Sense strand reverse transcript primer |
| ZNF280BY_ASRT | CGGCAGTTGAAGCGGCGTCC |  | Antisense strand reverse transcript primer |
| ZNF280BYqF | TTGTTCCCTCGGGCCTGCCT | 57 | Strand specific quantitative PCR forward primer |
| ZNF280BYqR | AAACTGCCACCGGTCCCCGT |  | Strand specific quantitative PCR reverse primer |
| ZNF280BRTF | CCCTTCAGGTATTTTTCAGC | 57 | RT-PCR forward primer for expression pattern |
| ZNF280BRTR | ATGTGATTCATAAACTTGAG |  | RT-PCR reverse primer for expression pattern |
| ZNF280AF | GAGGCGCATTTTAGAACGTG | 57 | RT-PCR forward primer for expression pattern |
| ZNF280AR | CCTCACAATGATGGATGCTG |  | RT-PCR reverse primer for expression pattern |
| ZNF280AY_1628F | ACATTTTGAACAGAGTGACC | 57 | RT-PCR forward primer for expression pattern |
| ZNF280AY_1900R | CTCCAGATAGACAATGAGGC |  | RT-PCR reverse primer for expression pattern |
| ZNF280AY_1049F | TCCTCATTGACATGCTGCAC | 57 | PCR |
| ZNF280AY_1574R | CCAACATCACTCCACTTACG |  | PCR |
| 18SrRNAqF | CACGGACAGGATTGACAGATTG | 57 | Strand specific quantitative PCR internal control Forward primer |
| 18SrRNAqR | CAAATCGCTCCACCAACTAAGA |  | Strand specific quantitative PCR internal control reverse primer |
| 18SrRNART | GCCTCACTAAACCATCCAATC |  | Reverse transcript primer |
