# Supplementary material for: ZNF280BY and ZNF280AY: autosome derived Y-chromosome gene families in Bovidae
Source: BMC Genomics. 2011 Jan 7;12:13. doi: 10.1186/1471-2164-12-13 (PMC3032696; doi:10.1186/1471-2164-12-13)
Supplement: Additional file 8 — Probe sequences for the testis section in situ hybridization. ZNF280BY sense and antisense cRNA probe sequences designed for the testis section in situ hybridization. Probe sequences for the bovine Protamine 1 (PRM1) gene (positive control) and LNE120 (negative control) are also included. [file 1471-2164-12-13-S8.DOCX]

**Supplementary Table 4** Probe sequences for the testis section *in-situ* hybridization.

| cRNA probe name | Sequence (5’- 3’) |
| --- | --- |
| ZNF280BY sense probe  ( For detection of antisense RNA) | GGAGGCATTGTTCCCTCGGGCCTGCCTCCTCAGACTTAACCCAGGGACTGTTTAGGTGCAGGTGAGCTGCCGGCAGTTCTGGCATCGCCGCCCACGGGGACCGGTGGCAGTTTATTTAAA |
| ZNF280BYantisense probe  (For detection of sense RNA) | TTTAAATAAACTGCCACCGGTCCCCGTGGGCGGCGATGCCAGAACTGCCGGCAGCTCACCTGCACCTAAACAGTCCCTGGGTTAAGTCTGAGGAGGCAGGCCCGAGGGAACAATGCCTCC |
| PRAMEY sense probe  (For detection of antisense RNA) | TGGCCACGCTGAGCAGGTTCCTGCCGCACCTGGGCCGGATGGGCAACCTGCGCCGGCTGCTGCTGTCTCGCATCCACATATTGCCACATACCACCCCGGACCAGGAGAACTGCGTCAACC |
| PRAMEY antisense probe  (For detection of sense RNA) | GGTTGACGCAGTTCTCCTGGTCCGGGGTGGTATGTGGCAATATGTGGATGCGAGACAGCAGCAGCCGGCGCAGGTTGCCCATCCGGCCCAGGTGCGGCAGGAACCTGCTCAGCGTGGCCA |
| PRM1 (Positive control) | GGCUGGUGACCUUUCAGGACAGGAGUGCGGUGGUCUUGCUACUGUGUGGUUACUGUCUUGUACACCUUAUGACGGUGUAGCGACGGCAGCACACUCUCCUCCUGCGCCUCCGACCAGAGC |
| LNE120 (Negative control) | UGCCUGCAAAGAUGAGGAGGGAUUGCAGCGUGUUUUUAAUGAGGUCAUCACGGGAUCCCAUGUGCGUGACGGACAUCGGGAAACGCCAAAGGAGAUUAUGUACCGAGGAAGAAUGUCGCU |
